# Diabetic Ketoacidosis and Hypertriglyceridemia-Induced Acute Pancreatitis Requiring Plasmapheresis: A Case Report of a Rare Presentation of Type 2 Diabetes Mellitus in Adults

**DOI:** 10.7759/cureus.52679

**Published:** 2024-01-21

**Authors:** João Lourinho, José Proença, Letícia Santos, Vanessa Leite, Sara Ramalho, Conceição Escarigo

**Affiliations:** 1 Infectious Disease, Hospital Garcia de Orta, Almada, PRT; 2 Internal Medicine, Hospital Garcia de Orta, Almada, PRT

**Keywords:** hypertrygliceridemic pancreatitis, lipid-lowering therapy, ketosis-prone type 2 diabetes, therapeutic plasmapheresis, hypertrygliceridemia, diabetic ketoacidosis (dka)

## Abstract

The triad of diabetic ketoacidosis, hypertriglyceridemia, and acute pancreatitis is a rare presentation of diabetes mellitus type 2 in adults. We report a case of a 41-year-old male who presented at the emergency department with a sudden onset of severe abdominal pain. Diagnosis of diabetic ketoacidosis, hypertriglyceridemia, and acute pancreatitis was made. Triglyceride level was 6056 mg/dL and glycated hemoglobin was 12.6%. Vigorous fluid therapy and continuous infusion of insulin were started, but due to maintained symptoms, a single plasmapheresis session was performed, with significant clinical improvement. This case emphasizes the diagnostic challenges of the triad and highlights the potential role of plasmapheresis in rapidly reducing triglyceride levels.

## Introduction

The well-known triad of diabetic ketoacidosis, hypertriglyceridemia, and acute pancreatitis, first described in 1997 as an “enigmatic triangle”, occurs in about 4% of patients with diabetic ketoacidosis, being particularly rare as a presentation of diabetes mellitus type 2 in adults [1,2].

The simultaneous diagnosis of these conditions has several important clinical implications, particularly regarding diagnosis, which is complicated by the clinical similarities between diabetic ketoacidosis and acute pancreatitis, and treatment, which must be more aggressive due to the great vascular volume depletion [1,3,4].

The classical therapeutic approach involves intravenous fluid therapy, insulin infusion, and antidyslipidemic drugs. In spite of that, in severe cases of hypertriglyceridemia, plasmapheresis can be used as a faster way to decrease triglyceride levels [5].

With this case report, we intend to highlight the diagnostic complexity, especially in a patient with no previous diagnosis of diabetes mellitus, and the need for a rapid approach, reviewing the possibility of using plasmapheresis to reduce triglyceridemia in such cases.

## Case presentation

A 41-year-old male, born in Pakistan, living in Portugal for the last three years, with no known diseases or regular medication, and no history of alcoholism nor lipid-rich diet, was admitted at the emergency department with sudden onset of severe abdominal pain, nonspecific general malaise, polyuria, polydipsia, and polyphagia, as well as a weight loss of 10 kg in the last month. On physical examination, he presented with hemodynamic and respiratory distress and abdominal tenderness.

**Figure 1:**
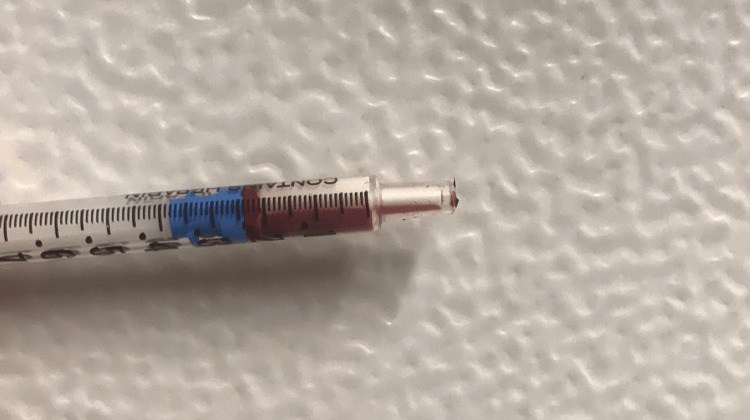
Arterial blood collected with a macroscopic lipid deposit.

Capillary blood glucose and ketonemia were elevated and arterial blood gas analysis showed metabolic acidemia with increased anion gap (Table 1). The arterial blood collected had a macroscopic lipid deposit (Figure 1). Laboratory investigations, as summarized in Table 1, revealed increased levels of amylase, while total bilirubin and alkaline phosphatase were within normal ranges. Triglycerides were markedly elevated and glycated hemoglobin was increased. An abdominopelvic computed tomography revealed acute edematous pancreatitis with an acute inflammatory collection, without necrosis.

**Table 1:** Summary of laboratory investigations and reference ranges.

Laboratory tests	Initial results	Reference range
Arterial blood gas		
pH	7.284	7.35-7.45
CO_^2^_ partial pressure	26.6 mmHg	32.0-48.0 mmol/L
HCO_3_^-^	12.3 mmol/L	22-32 mmol/L
Anion gap	15 mmol/L	4-12 mmol/L
Blood tests		
Capillary blood glucose	323 mg/dL	
Ketonemia	6.4 mmol/L	<0.5 mmol/L
Glycated hemoglobin	12.6%	4.8-6.0%
Amylase	294 IU/L	<100 IU/L
Total bilirubin	0.5 mg/dL	<1.2 mg/dL
Alkaline phosphatase	105 IU/L	40-129 IU/L
Triglycerides	6056 mg/dL	<150 mg/dL
C-peptide value	13.60 ng/mL	0.80-3.50 ng/mL
Autoimmunity tests		
Anti-islet antibody	Negative	
Anti-GAD antibody	Negative	
Anti-insulin antibody	Negative	

**Figure 2:**
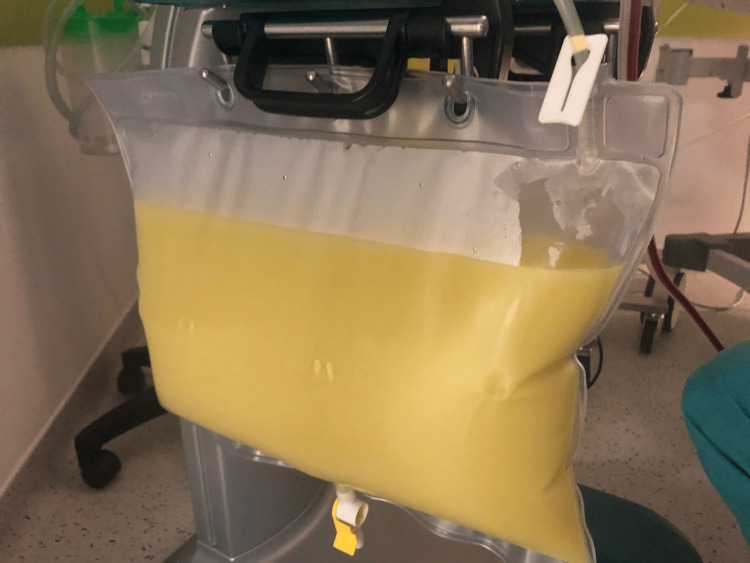
Lipid-rich plasma filtered by plasmapheresis.

Intravenous continuous infusion of regular insulin, vigorous fluid therapy, and intravenous potassium replacement were started, with improvement in metabolic acidemia. However, the patient maintained significant abdominal pain with poor response to analgesia. Considering the clinical severity and high concentration of triglycerides, it was decided to proceed with the treatment of plasmapheresis and admit the patient to the intensive care unit for further management. Approximately 28 hours after admission, one plasmapheresis session was performed without complications, resulting in a significant drop in the triglyceride level to 223 mg/dL and a pronounced clinical improvement (Figure 2).

The patient evolved favorably with the normalization of pH and anion gap. There were no local or systemic complications of acute pancreatitis. The patient was discharged from the hospital on the 13th day of hospitalization and medicated with subcutaneous insulin and dual antidyslipidemic therapy. At discharge, the triglyceride level was within the normal range.

Three months after discharge, the glycated hemoglobin value dropped to 6.6%. Regarding the etiological study, the C-peptide value was elevated and the anti-islet, anti-GAD, and anti-insulin antibodies were negative, and diagnosis of type 2 diabetes was confirmed. After discharge, glycemic control was achieved with only oral antidiabetic therapy.

The patient had no new admissions to the emergency department and has remained asymptomatic in the two years after hospitalization.

## Discussion

We report the case of a 41-year-old male with an initial presentation of type 2 diabetes mellitus with severe abdominal pain from diabetic ketoacidosis and hypertriglyceridemia-induced acute pancreatitis.

Diabetic ketoacidosis is traditionally described as a complication of type 1 diabetes mellitus, but it can also be found in type 2 diabetes mellitus, most frequently in a condition known as ketosis-prone diabetes mellitus, where there is an acute and temporary deficiency in insulin secretion and sensitivity [6]. The recognition of ketosis-prone type 2 diabetes mellitus is important as it often allows the transition from insulin to oral antidiabetics over time, with improved quality of life, as happened in our case [7].

In diabetic ketoacidosis, insulin deficiency causes lipolysis of adipose tissue and decreases the removal of circulating lipoproteins by peripheral tissues. These mechanisms increase triglycerides in the blood [1]. Hypertriglyceridemia causes acute pancreatitis by releasing free fatty acids, which are toxic to the endothelium and acinar cells of the pancreas. Hypertriglyceridemia-induced acute pancreatitis can, in turn, lead to acute pancreatic beta-cell dysfunction, resulting in transient insulin deficiency, which can lead to hyperglycemia [8]. Also, a severe episode of diabetic ketoacidosis with hyperlipidemia is more likely to be linked with acute pancreatitis [9]. In our case, as in other previously described cases, we assumed diabetic ketoacidosis as the precipitating event for the worsening of hypertriglyceridemia and, consequently, for the onset of acute pancreatitis, taking into account the substantial increase in glycated hemoglobin [2].

The treatment of diabetic ketoacidosis involves intravenous fluid therapy, insulin therapy, and treatment of the precipitating cause [10]. The diagnosis of acute pancreatitis concomitant with diabetic ketoacidosis is important for a more adequate therapeutic approach, namely the need for more aggressive intravenous fluid therapy, taking into account the great depletion of intravascular volume and the evaluation of possible local and systemic complications of pancreatitis [1].

Wan J et al. have demonstrated that, together with other prognostic factors, hypertriglyceridemia may be associated with greater severity and mortality in acute pancreatitis [11]. Plasmapheresis is very effective in reducing triglyceridemia [12]. It is indicated in medical emergencies, such as acute pancreatitis due to hypertriglyceridemia when the triglyceride concentration is greater than 1000 mg/dL and is contemplated in the guidelines of the American Society for Apheresis [5,13]. In our case, taking into account the clinical severity, namely the difficulty in controlling the abdominal pain and the high concentration of triglycerides, we chose to perform plasmapheresis with a large drop in the triglycerides level and symptomatic improvement. It is difficult to assess whether the control of symptoms was due to plasmapheresis or to the other therapies performed. At the same time, it is uncertain whether triglycerides would not decrease as rapidly without the use of plasmapheresis, as described in other cases [2]. To our knowledge, this is the only case in adults of the triad of diabetic ketoacidosis, hypertriglyceridemia, and acute pancreatitis where plasmapheresis was performed to reduce the triglyceride level.

Prompt reduction of severe hypertriglyceridemia by apheresis removes the causative agent for pancreatic damage, providing a more effective approach [13]. Tailoring treatment to the unique aspects of the patient’s condition enhances the likelihood of a positive outcome.

## Conclusions

We conclude that the triad of diabetic ketoacidosis, hypertriglyceridemia, and acute pancreatitis is a rare presentation of type 2 diabetes mellitus in adults. Diabetic ketoacidosis and acute pancreatitis are medical emergencies, and the combination of the three entities emphasizes the need for rapid therapy.

Hypertriglyceridemia is an independent poor prognostic factor in acute pancreatitis, and plasmapheresis may be used to reduce the triglyceride level rapidly. Despite this, there is no consensus on the use of plasmapheresis in its management. Further studies comparing conventional therapy and plasmapheresis in the treatment of hypertriglyceridemia seem to be necessary, in order to better define the patients who benefit the most from plasmapheresis.
